# Ideological polarization during a pandemic: Tracking the alignment of attitudes toward COVID containment policies and left-right self-identification

**DOI:** 10.3389/fsoc.2022.958672

**Published:** 2022-10-28

**Authors:** Stephan Dochow-Sondershaus

**Affiliations:** Freie Universität Berlin, Institute of Sociology, Berlin, Germany

**Keywords:** issue alignment, COVID attitudes, left-right self-identification, polarization, COVID containment policies, ideology, party sorting

## Abstract

Research on opinion polarization has focused on growing divides in positions toward political issues between the more politically and ideologically engaged parts of the population. However, it is fundamentally difficult to track the alignment process between ideological group identity and issue positions because classically controversial political issues are already strongly associated with ideological or partisan identity. This study uses the COVID pandemic as an unique opportunity to investigate polarizing trends in the population. Pandemic management policies were not a politicized issue before COVID, but became strongly contested after governments all across the world initiated policies to contain the pandemic. We use data from the Austrian Corona Panel Project (ACPP) to track trajectories in attitudes toward current COVID measures over the course of more than a year of the pandemic. We differentiate individuals by their ideological self-identity as measured by left-right self-placement. Results suggest that all ideological groups viewed the containment measures as similarly appropriate in the very beginning. However, already in the first weeks, individuals who identify as right-wing increasingly viewed the policies as too extreme, whereas centrists and left-wing identifiers viewed them as appropriate. Opinion differences between left-wing and right-wing identifiers solidified over the course of the pandemic, while centrists fluctuated between left and right self-identifiers. However, at the end of our observation period, there are signs of convergence between all groups. We discuss these findings from the perspective of theoretical models of opinion polarization and suggest that polarization dynamics are likely to stop when the political context (salience of certain issues and concrete material threats) changes.

## Introduction

There is an ongoing debate in sociology and the political sciences about the extent and breadth of polarization in Western democratic societies (DiMaggio et al., 1996; DellaPosta and Macy, 2015; McCarty, 2019). However, when it comes to attitudinal divides between politically engaged groups that share broad ideological similarities, the evidence consistently shows polarizing trends. A prime example is partisan polarization in the US, the rising differences in policy positions and growing animosity between supporters of the Republicans and Democrats (Fiorina, 2017; McCarty, 2019). Similar arguments have been made for European countries (Westwood et al., 2018; Flores et al., 2022).

This article investigates the dynamics of ideological group polarization, the increasing differences in substantive policy attitudes between groups that ascribe to certain ideological labels, in the specific historical context of the COVID pandemic. In particular, we analyze the potential evolving alignment between left-right self-positioning and individuals' positions toward current COVID policies^1^.

It seems obvious that individuals who ascribe to different ideologies have different attitudes toward the politicized aspects of social life (McCarty, 2019). Indeed, this might be considered a necessary part of a functioning pluralist democracy. However, if the politically engaged and active parts of society hold incompatible attitudes on a wide variety of issues, or one issue that is extremely politicized, the chances of political consensus might vanish. Furthermore, the existence of homogenous ideological camps might lead political actors of either camp to disengage from persuasion and start preaching to the choir because the other side is deemed unreachable (McCarty, 2019). This might lead to the solidification of already existing social bubbles. Most importantly, while these bubbles might be initially constrained to individuals engaged in politics, examples such as the USA (Iyengar et al., 2019) and Hungary (Vegetti, 2019) show that polarized elite level discourse can lead to polarized societies and worrisome consequences for social cohesion at large.

These potential threats to social cohesion are particularly apparent in times of a pandemic, where a certain normative consensus is required for both political decisions making and in interpersonal social encounters. Political decisions have to be made quickly and revised as epidemiological research progresses, requiring consensus about facts concerning the pandemic and the usefulness of certain measures among political actors. Furthermore, individuals require their neighbors' or family members' cooperation in social situations in the face of epidemiological dangers. Social situations where some individuals enforce and follow state policies, while others oppose them, will results in uncertainty and coordination dilemmas. This might be the case even if outright rejection of certain policies is only expressed by small parts of the population. In contrast to other political disputes, coordination dilemmas in pandemic situations are likely to occur in the everyday life of individuals, for example, when family members diverge in their compliance with mask mandates.

This paper contributes both to the literature on ideological group polarization and theoretical models of opinion polarization by offering a temporally fine-grained analysis of the dynamics of opinion divergence between ideological groups. The COVID pandemic presents an unique opportunity to study how political positions align with ideological self-identity because COVID entered political discourse suddenly and pandemic management was not a politicized issue before COVID. Thus, it is safe to assume that there are no prior affinities between ideological self-descriptions and attitudes toward pandemic management. Furthermore, the study is conducted in Austria, a country with a long tradition of right-wing populism (Mudde and Rovira Kaltwasser, 2013) and where right-wing actors strongly used the pandemic for political purposes. These particularities of the case, together with fine-grained longitudinal data, allow us to study an ideal case where social influence by the own ideological group should be have a major influence on individual attitudes, and thus, polarization should escalate according to most models of opinion polarization.

The topic of differences between ideological groups is strongly related to research on partisan polarization. Partisan identity is an important political group marker in the US two-party system. In European multi-party contexts, such as the Austrian context, ideological self-identification might serve a similar function as partisanship in the US. Indeed, Europeans often do not have durable party affiliations or voting behavior (van der Meer et al., 2015), whereas ideological self-identification tends to be more stable (Peterson et al., 2020). And while partyism has been observed in the European context, it is strongly conditional on the ideological distance that partisans perceive to the other party (Westwood et al., 2018).

## Processes of ideological group polarization

This article views ideological group membership as an indicator of two aspects that play an active role in societal polarization dynamics. The first aspect is related to the content of the ideology, its principles and the attitudinal priors that individuals derive from these principles. Thus, ideology might serve as a set of attitudinal heuristics in the face of complex social problems (Lütjen, 2020). The second aspect are the group-level social implications of affiliating with the same ideology. Individuals in the same ideological groups might share similar or related political information channels, encounter similar arguments in their interpersonal social influence networks and share a social identity (Mason, 2018). More generally, they are positioned in the same realm of social influence.

The probably most intuitive individual-level mechanism how ideological group membership affects opinion formation is that individuals process the same information differently based on their *ideological priors*, which leads them to embrace different political opinions (Newman et al., 2018). For example, Lütjen (2020) argues that polarization is a predictable outcome of individuals' need to filter information in times of increasing complexity. Positions on the left-right scale are commonly theorized to stem from political attitudes along two axes: an economic and a socio-cultural axis (Lachat, 2018). On the economic axis, the left pole stands for pro-state, progressive and interventionist positions, while the right pole stands for market-liberalism and self-responsibility. On the socio-cultural axis, the left pole stands for culturally liberal, social justice, pro-immigration positions, while the right pole stands for conservative, authoritarian, law-and-order positions (de Vries et al., 2013). Ideology might lead to initial attitudinal affinities toward COVID policies that get strengthened in the course of the pandemic *via* the social influence mechanisms outlined in the following. For example, it might be reasonable to assume that individuals who position themselves on the right might be more inclined to be in opposition to COVID policies, because they view them as an infringement of individual liberty.

Beyond the psychological content of ideology, *social influence* is the most well-studied mechanism in theoretical models of opinion polarization (DellaPosta and Macy, 2015). One widely shared assumption among these models is that individuals adopt information more readily from individuals who are like them in many respects, an assumption based on empirical evidence of ubiquitous homophily in human social networks (McPherson et al., 2001). Additionally assuming that actors distance themselves from others with dissimilar opinions (DellaPosta et al., 2015; Axelrod et al., 2021) or that actors exchange arguments with similar others, which in turn reinforces their worldview (Mäs and Flache, 2013) leads to polarized opinion landscapes: initial attitudinal affinities within groups are re-enforced by social influence and lead to escalating opinion divergence over time.

Ideological group membership likely structures social influence networks by determining the sources from which individuals obtain information on newly emerging political issues. Real-world social influence can be manifold. First, individuals might discuss political issues with persons in their personal networks, which are likely segregated by political identity (Jiang et al., 2020). Particularly when it comes to newly emerging, politically salient issues they might form their opinion in discussions with their ideologically like-minded peers. Furthermore, there is also evidence for active distancing between ideological groups in the US (Iyengar et al., 2019) and in Europe (Westwood et al., 2018).

Second, ideological groups might share similar information channels in the form of the media channels that they consume and the public figures and social media accounts that are prominent in certain ideological circles. For example, research has shown that political elite communication on COVID differed markedly between ideological groups (Green et al., 2020) and Twitter networks were highly politically polarized (Jiang et al., 2020). Thus, this article assumes that ideological groups form a realm of shared media influence. Importantly, we do not assume that each individual in each group consumes the exact same media channels, but that there is a certain affinity toward certain outlets and opinion makers (Prior, 2013; Cardenal et al., 2019), which leads to a propagation of specific ideas about COVID through these realms over time. Thus, the reasoning underlying this paper acknowledges that ideologies are best thought of as diverse coalition of individuals that do not necessarily share the same opinion on all issues (Noel, 2013, p. 19), but still are subject to the same talking points (Mäs and Flache, 2013).

Note that we stress the social influence mechanism over the ideological prior mechanism because in the presence of previously unpoliticized issues, the exact reaction on how certain ideologies incorporate their views on political issues into a consistent worldview are often unpredictable (Macy et al., 2019). Indeed, from a perspective that focuses only on the consistency of ideological content, both left and right ideological principles lend themselves to support either strict or laissez-faire COVID containment strategies. From a right-wing conservative perspective, the state could ensure law-and-order and the health of the native population by prohibiting large outbreaks. From the left, state policies against COVID outbreaks could be justified by the necessity to help vulnerable groups. On the other hand, both the right and the left could have opposed strong state interventionism by criticizing the restrictions to individual freedom that go along with containment policies, be it economic freedoms (right) or freedoms of movement and cultural expression (left).

Investigating the perception of state measures during the COVID pandemic presents a unique opportunity to study social influence in a most likely scenario. This is for two reasons. First, pandemic policies were not politicized before the pandemic, which leads to the plausible assumption that positions on pandemic management were not part of the traditional political issues that make up ideologies. As we can see in later analyzes, individuals from all over the left-right spectrum had similar attitudes toward anti-COVID measures in the very beginning of the pandemic. Indeed, COVID is an interesting case because one could easily imagine an unpolitical, technocratic way of debating the pandemic based on established facts from epidemiological and medical research. As a contrary example, traditional left-right issues such as immigration are deeply entrenched in individuals' ideological self-perception, and social influence might already have largely played its role when researchers begin to study ideological group polarization. Thus, the pandemic allows us to follow the dynamics of polarization from the early beginning.

Second, in the very first weeks of the pandemic, pandemic management emerged as a strongly politicized issue (Hart et al., 2020; Flores et al., 2022). While political echo chambers are never perfect (Cardenal et al., 2019), there is strong reason to expect that individuals who ascribe to the left or right first seek information from ideological peers or their known information networks when an issue suddenly enters the political sphere. Research suggests that elite influence can lead to opinion differences, even for issues that were previously non-divisive (Levy Yeyati et al., 2020). This is exactly the case with COVID, an issue that was not covered largely by political actors before the pandemic, leaving amble scope for influence of opinion makers after the onset of the pandemic (Flores et al., 2022). Furthermore, the need to reduce complexity (Lütjen, 2020) works in tandem with social influence mechanisms: Particularly at the beginning of a public health crisis, a situation characterized by high uncertainty, we should expect individuals who are politically engaged to cling to their own group when forming their policy positions.

From these premises and empirical findings, we derive our first hypothesis: We expect that ideological groups should increasingly grow apart from each other in their assessment of the appropriateness of COVID measures in the course of the pandemic (Hypothesis 1: *Repelling Curves Hypothesis*). This should be primarily the case for differences between high-identifiers, and less strong for individuals who would describe themselves as centrists (Jewitt and Goren, 2016).

Note that the Repelling Curves Hypothesis is agnostic about which ideological group develops which position toward COVID measures. It just states that group differences get larger over time. However, in the Austrian context it is possible to make more precise predictions when considering the messaging of political elites. In European and US right-wing actors embraced massaging that were critical of most of the COVID containment strategies (Jungkunz, 2021; Froio, 2022). In Austria in particular, the right-wing populist party FPÖ first took a positive stance toward strict containment measures, but changed to an extremely skeptical stance within the very first weeks of the pandemic (Mellacher, 2020; Thiele, 2022). Elite messaging likely has behavioral consequences in the public. For example, in Austria, areas with high FPÖ vote shares had higher COVID deaths (Mellacher, 2020). And even in Italy, a country severely hit by the pandemic, provinces with higher right-wing vote show lower rates of compliance with social distancing orders (Barbieri and Bonini, 2021). Similarly, Jungkunz (2021) argues that affective polarization between partisans of the German right-wing populist AfD and other parties increased substantially during the pandemic. While there might be certain segments on the left that also embraced positions against current COVID measures, for example more esoteric, new age left (Frei and Nachtwey, 2022), the most pronounced institutional protest certainly came from right-wing actors.

These previous findings lead to a second, more directed hypothesis. We predict that right-wing identifiers should experience a particularly strong increase in their opposition to current COVID containment measures which sets them apart from the other groups in a distinctive way (Hypothesis 2: *Right-Wing Outliers Hypothesis*).

## Data and methods

This study uses the Austrian Corona Panel (ACPP Scientific Use File, version 4, published 2021-10-08) (Kittel et al., 2020)^2^. The ACPP fielded first in March 27, 2020 (31 days after the first registered COVID patient in February 25, 2020). The ACPP is an online survey that is conducted in a sample drawn from a pre-existing online access panel run by Marketagent, Austria. Respondents were chosen based on quota sampling by age, gender, region (Bundesland), municipality size, and educational level based on official population statistics. The data have been analyzed for quality and representativity in previous publications (Aschauer et al., 2022). The Scientific Use File contains data until July 2, 2021 with 24 waves in total. Thus, respondents are surveyed frequently, often weekly, during the period of observation. Furthermore, regular refreshment samples ensure that the sample size in each wave is about 1,500. We include all respondents who were sampled before wave 11 or June 3, 2020 (see below).

Austria pursued similar COVID containment strategies to many of its neighboring countries. When the pandemic hit Austria in March 2020, Austria's government, run by Federal Chancellor Sebastian Kurz from the center right party ÖVP, mandated a short, but severe lockdown. Several municipalities, which are well-known skiing resorts, were quarantined^3^. In March and April, first mask mandates were introduced. In Spring 2020, the declining COVID cases allowed for a wide-ranging lift of many policies that limited movement and public gatherings. This phase of relatively few restrictions lasted until the autumn of 2020, when another period of state-wide lockdowns and other restrictions began. Throughout the winter of 2020/2021, there were multiple restrictions which were lifted when COVID cases declined in May 2021. When reviewing the main results, we will outline the broad historical events that matter for the interpretation of our results.

Using online access panels in prone to problems of representativity, which has consequences for the interpretation of the results. First, all results are only generalizable to the population of individuals with internet access. Second, even when using quota sampling to reproduce characteristics of the overall population, there might be unknown factors that influence taking part in an online survey and the outcome of interest. To take one step in the direction of decreasing bias, all analyzes are weighted by the wave-specific demographic and political survey weights. The demographic weights ensure that the sample corresponds to marginal frequencies of demographic variables in the Austrian census. The political weights are based on retrospective information from a question that asks for the party that respondents voted for in the 2019 national election to weight the sample such that the marginal distribution of voting behavior in the ACPP sample matches the official results of the 2019 national election. Weighting increases the confidence intervals substantially compared to un-weighted analyzes, but the overall conclusions are similar in both analyzes (for un-weighted analyzes, see Supplementary Figure S2 and Table S2).

Our main outcome variable is based on a survey item that asks respondents to assess the appropriateness of current COVID policies. The question reads “Do you consider the response of the Austrian government to the coronavirus to be insufficient, appropriate or too extreme?.” Respondents answered on an ordinal 5-point scale with response options “not sufficient at all,” “rather sufficient,” “appropriate,” “rather too extreme,” and “too extreme.” There are several particularities of this item that require elaboration. First, note that responses to this item are highly influenced by the current policies that are in place. Thus, responses should be interpreted in the specific context they were obtained, which we provide when reviewing the results. Since policies change with the pandemic situation, we should see volatility in the average responses to this item. Second, the population-wide average response to this item cannot be taken as an indication of the level of social cohesion in the population. While widespread opposition to COVID measures indicates a conflict between politicians and the public, this does not necessarily strain personal networks. Third, however, group differences in responses to this item are highly indicative of polarization. This is because one group behaving under the impression that the policies are adequate, while another group opposes the policies, exactly leads to the type of coordination dilemmas and interpersonal unease that we outlined in the introduction. Furthermore, the target of the item (current COVID measures) is salient in respondents' perception and, thus, comes close to how they see the world in the moment they took part in the survey.

Our main independent variable is ideological self-identification at the beginning of the pandemic. The variable is measured in a specific questionnaire that is provided to each new participant and asks about general socio-demographic information. The item wording is “In politics, one speaks again and again of “left” and “right.” Where would you place yourself on this scale, with 0 meaning left and 10 meaning right?.” We recode 0, 1, and 2 to “left”; 3 and 4 to “center left,” 5 to “center,” 6 and 7 to “center right” and 8, 9, and 10 to “right.” This results in a categorical variable distinguishing five groups, which we treat as time-constant. We restrict our baseline sample of individuals to those whose ideological self-identification was measured before wave 11 or June 4, 2020. This step is important because early self-identification is better able to capture social influence networks and ideological priors *before* the pandemic than later measures (the next wave after wave 11 where the same item was asked is wave 20). This is because individuals might switch affiliations in the course of the pandemic, maybe even because of their newly formed attitudes toward pandemic management. This sample restriction also means that our sample of analysis only includes individuals who entered the ACPP before wave 11.

**Figure 1:**
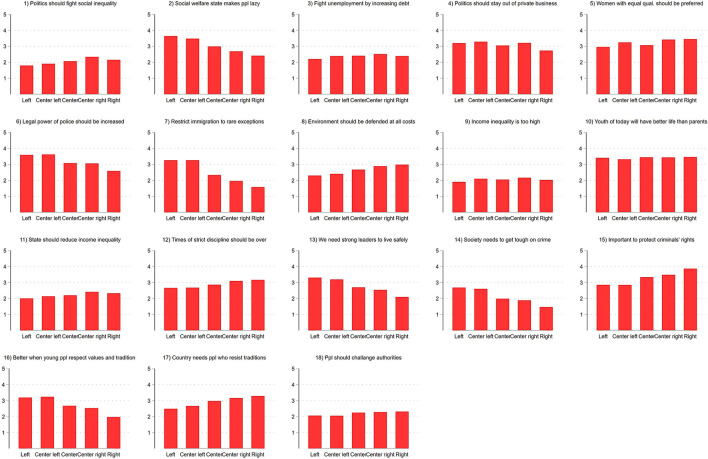
Average position on several political issues by ideological group. Average response to items that asks whether certain statements about political topics apply. Response categories range from 1 “completely applies” to 5 “does not apply at all.” Items were asked in wave 5 (April 24, 2020–April 29, 2020).

Since the meaning of left and right differs between national contexts, Figure 1 provides an overview of associations between left-right identification and responses to items asking about political positions on several issues. We can clearly see that left-right is associated the most with attitudes about law-and-order (items 6, 12, 13, 14, and 15), immigration (item 7) and honoring tradition (16, 17). Right-wing identifiers in our sample are more likely to attest a deficit in values and traditions and that immigration to Austria should be restricted. In contrast, the association between left-right self-placement and economic issue positions is small. For example, there is almost no difference between ideological groups in their response to whether politics should fight social inequality, the state should fight unemployment by increasing debt or the state should intervene less in the economy. One exception is that right-wing identifiers are more likely to state that social welfare state makes individuals lazy.

Because the development of attitudes toward COVID measures is likely to vary in a wave-like fashion with the strengthening and weakening of measures to curtail the pandemic, we use restricted cubic spline functions to model the non-linear relationship between interview date and attitudes (Durrleman and Simon, 1989). We place knots evenly at 40, 100, 200, 300, and 420 days after the first Corona infection in Austria (February 25, 2020).

For descriptive statistics at different time points, please refer to Supplementary Table S1.

**Figure 2:**
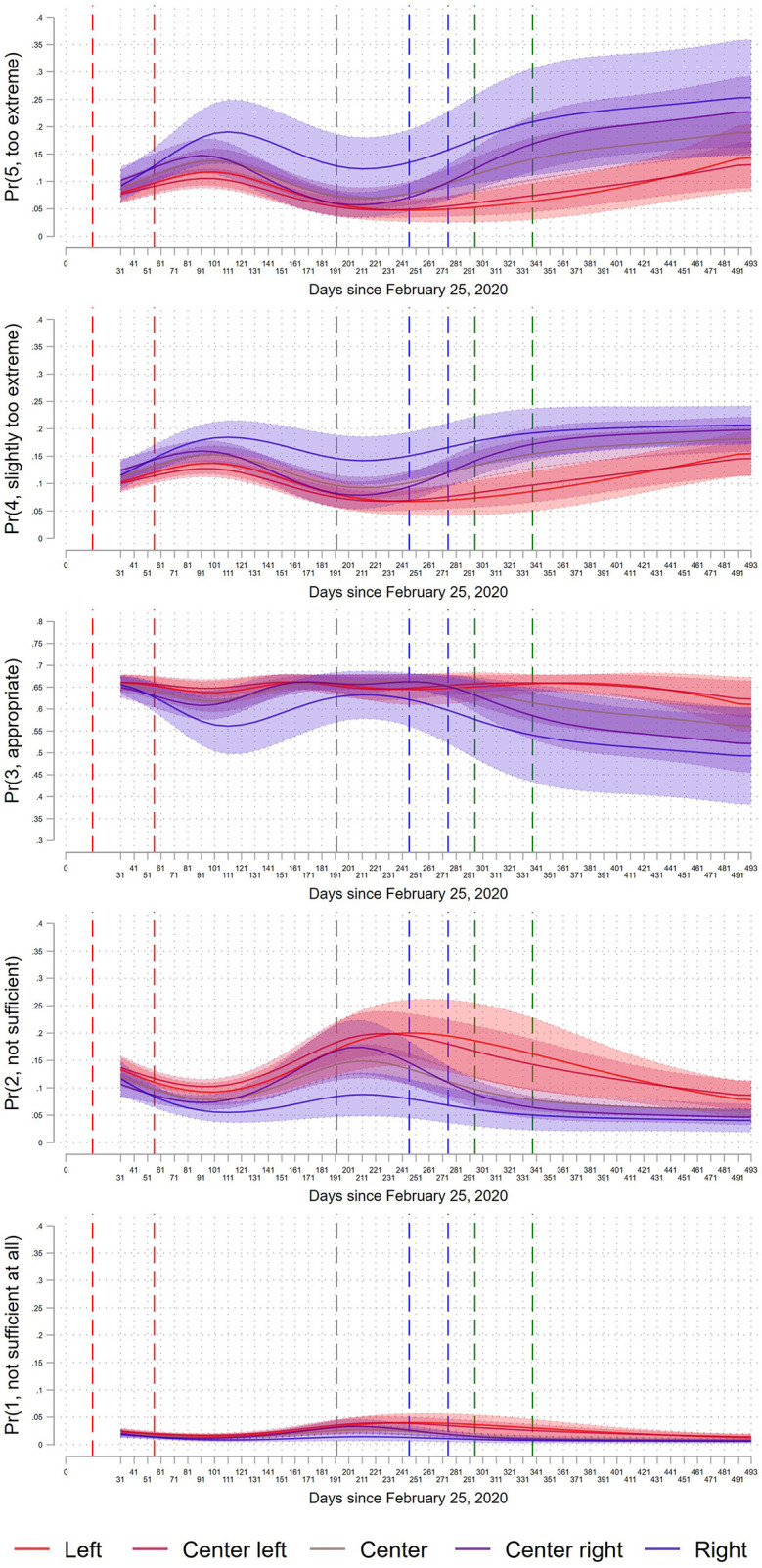
Predicted probabilities of responses to “Do you consider the response of the Austrian government to the coronavirus to be insufficient, appropriate or too extreme?” on a five-point scale. Probabilities derived from ordered logistic regression models, conditional on left-right self-placement at the beginning of the pandemic. Model adjusts for time-stable socio-demographic variables. Vertical lines show historical events: red = beginning/end of first lockdown, gray = introduction of strict mask mandates, blue = beginning/end of second lockdown, green = beginning/end of third lockdown. Areas around curves indicate 95% confidence intervals.

Our main results (Figure 2) are based on predicted probabilities derived from ordered logistic regressions. We regress our outcome variable on the previously mentioned indicators of ideology and time, and their interaction. We also include the following socio-demographic variables: age, education, sex, regional dummies (Bundesland), whether respondents have access to a balcony or garden, and whether respondents have preconditions that make them vulnerable to COVID. These variables can also be seen in Supplementary Table S1. We also include an interaction between education and time because education is an important predictor of policy attitudes and its effect might vary with the pandemic.

Average predicted probabilities for each ideological group and time point are calculated using Stata's margins command (Stata version 17.0). We set the time and ideological group variables to their respective value of interest, while leaving the remaining covariates at their unit-specific values to derive individual outcome probabilities. We then average these individual predicted probabilities over ideological group and time (Mood, 2010).

Note that our results are robust to using model specifications without control variables (see Supplementary Figure S1) and without weights (see Supplementary Figure S2). Linear Growth Curve Models also lead to similar results (see Supplementary Figure S3). For the full regression tables of all models, please refer to Supplementary Table S2. We discuss the usage of the more flexible multinomial logistic regressions at the end of the results section.

Finally, all results below are adjusted for potential panel attrition by weighting with the inverse probability of staying in the sample (Robins et al., 2000). The probability to stay in the sample is modeled in a logistic regression model as a function of the previously measured response a respondent gave to the outcome variable (and additional time-stable demographic variables to stabilize the weights, see Robins et al., 2000). Thus, we adjust for potential drop-out in case individuals who grow wary of the COVID measures also develop a distrust toward scientists, which might affect their participation in scientific surveys.

## Results

Figure 2 depicts trends in the average predicted probabilities of responding that current COVID policies are “too extreme” (5), “slightly too extreme” (4), appropriate (3), “not sufficient” (2) and “not sufficient at all” (1) by respondents' ideological self-identification. The predicted probabilities are derived from an ordered logistic regression model, controlling for socio-demographic variables and weighted for both panel attrition and socio-demographic and political weights (see above).

Before turning to differences between ideological groups, it is worth mentioning that there are general results that hold across groups. Most importantly, the majority of respondents in all groups consider current COVID measures appropriate throughout the observation period (see third panel in Figure 2). This finding holds even for groups which display decreasing trends in the probability to respond “appropriate.” For example, half of right-wing identifiers still respond “appropriate” at the end of the study period, despite the fact that they do so less than at the beginning of the study. Furthermore, the response category “not sufficient at all” was rarely chosen by respondents from all five ideological groups.

To discuss differences between the five ideological groups, it is helpful to decompose the overall trends into four periods. In the first weeks of the pandemic, during the first lockdown (beginning and end are depicted by vertical dashed red lines), we find a consensus among all ideological groups. The first lockdown was characterized by strict containment policies, including closing of businesses and quarantine measures in certain states. Still, all response options were chosen with similar probabilities in the five groups: “Appropriate” with about 0.65 probability and “not sufficient,” “slightly too extreme” and “too extreme” each with about 0.1 probability.

The following period, from May to the summer of 2020 shows a general consensus among most groups and a take-off phase for right-wing identifiers. This period was characterized by a low number of restrictions and re-opening of many locations of social life. There are two interesting aspects in Figure 2. First, most of the political spectrum from center-right to left is in consensus, showing similar probabilities for each response category. For example, 100 days after the first COVID cases in Austria, differences between left identifiers and all groups except right identifiers in responding “too extreme” range between −1% point (center left) and 3% points (center right) and these differences are all non-significant by conventional standards. Those groups that experienced slight increases in responding “too extreme” or “slightly too extreme” during the first lockdown mostly fall back to their initial level.

Second, however, right-wing identifiers depart from this general picture by showing increased skepticism toward the COVID measures. Already shortly after the first lockdown, right-wing identifiers increasingly responded “too extreme” and “slightly too extreme” and decreasingly chose “appropriate.” After the first lockdown (second dashed red line), we can see continuations of these trend (at 100 days, the difference between right and left identifiers in responding “too extreme” is 7% points, *p* = 0.015). Even more interesting is that this increasing skepticism solidifies within right identifiers: the higher probability of choosing “too extreme” among this group remains constant over the whole course of the pandemic. This solidification of opposition to COVID measures happens during a time when there was no large-scale state repression to uphold COVID requirements. This is an important finding because it shows that attitudes toward the COVID measures got divorced from material reality in parts of right-wing identifiers: even though restrictions were kept relatively minimal in the summer of 2020, opposition among right-wing identifiers remains higher than in the other groups.

The picture changes again at the beginning of the second wave in November 2020 and the introduction of stricter and encompassing mask mandates (September 14, 2020; gray dashed line) and the second lockdown (blue dashed lines) onwards into the year 2021. Whereas, right-wing identifiers were the “outliers” in the aftermath of the first lockdown, this period is characterized by solidification on the left and an intensifying “left vs. the rest” scenario. Center-right identifiers and centrists show increasing probabilities to choose “too extreme” and “slightly too extreme.” In contrast, center left and left identifiers follow a different trajectory by maintaining their low probability of choosing “too extreme” and “slightly too extreme,” but increasing their probability of responding “not sufficient.” This leads a growing distance between, not only right-wing identifiers and (center) left identifiers, but also between (center) left identifiers and centrists. For example, at 330 days after the first case in Austria, there is no difference between left identifiers and center-left identifiers, but there are significant differences between left identifiers and center (7% point difference, *p* = 0.002), center-right (10% point difference, *p* < 0.001) and right (14% point difference, *p* = 0.005).

The final period is characterized by slow convergence in all groups. Particularly after the third lockdown (green vertical lines), which began in December 27, 2020 and lasted until February 2, 2021; centrists, center-right identifiers and right identifiers seem to experience a limit to their increasing opposition to COVID measures, whereas left and center-left identifiers show increasing trends in responding “slightly too extreme” and “too extreme,” and a strong decrease in the probability to respond “not sufficient.” While there were still regional lockdowns in the first half of 2021, there were also multiple signs of normalization: test kits were widely available, the rate of vaccinated Austrians rose steadily and more and more containment policies were rolled back^4^.

It is important to note that choosing the parsimonious ordered logistic regression might miss some aspects of the data compared to more flexible data fitting approaches. Thus, we contrasted the results from the ordered logistic regression to results of a multinomial logistic regression (see Supplementary Figure S4). Ordered logistic regression makes the proportional odds assumption which allows to estimate predicted probabilities of ordinal outcomes with relatively few parameters. In contrast, multinomial logistic regression does not make this assumption but is more data intensive and requires substantially more parameters to be estimated. Thus, there is a tradeoff between parsimony and “letting the data speak for itself.” Since our data is limited with respect to case numbers, particularly in the extreme ideological groups, we chose the simpler ordered logistic regression for our main results. The results from the multinomial logistic regression in Supplementary Figure S4 lead to substantially similar conclusions about the dynamics of polarization. In particular, the early right-wing take-off phase is visible as a substantial increase in “too extreme” responses among right-wing identifiers. Furthermore, the left-vs.-the-rest phase is visible in an increasing probability to respond “not sufficient” among left and center-left identifiers. Finally, the final convergence phase is also visible in Supplementary Figure S4, albeit slightly differently than in Figure 2: At the end of the observation period, all groups together increase their probability to respond “appropriate,” and left-wing identifiers increase their probability to choose “too extreme.”

## Discussion of results and conclusions

These results paint a complex picture about the emergence of ideological polarization. On the one hand, some periods show clearly polarizing trends. Hypothesis 2, which suggests that right-wing identifiers adopt especially critical stances toward COVID measures, is confirmed for the first part of our observation period from the first lockdown until the second lockdown. However, around the time of the introduction of the most stringent mask mandates onward (gray vertical line), centrists and center-right identifiers began to distance themselves from the left and followed right identifiers' trajectory toward more skepticism toward the COVID policies. This leads to a new constellation in the later stage of the pandemic, where left-wing identifiers are most distant to the other groups. Thus, we can discern two periods where the largest differences are driven by different groups. The first is driven by the early take-off of perceiving COVID measures as “too extreme” by right-wing identifiers, the second is driven by left-wing identifiers who deem COVID policies insufficient. A further interesting result is the behavior of centrists. In our data, centrist individuals maintain positions between the two ideological poles, but first align with the left and later follow the right by increasing their weariness of COVID policies.

These findings parallel predictions of theoretical models of polarization (and our Repelling Curves Hypothesis). Social influence processes in homophilic networks (DellaPosta and Macy, 2015) would lead to a growing divide between groups which are segregated in their social exchange and information networks. This is what we assumed for left and right identifiers, and, indeed, the results show that the divide between those two groups is largest, stays largest and increases in certain time periods These results are also in line with previous research, which found polarization in positions on COVID policies between groups that ascribe to different parties (Mellacher, 2020; Jungkunz, 2021; Flores et al., 2022). We extend these results to groups of left-right self-identifiers and provide a detailed description of opinion dynamics. Our results are also consistent with research on affective polarization (Westwood et al., 2018; Iyengar et al., 2019; Jungkunz, 2021): The fact that polarization around COVID occurred so rapidly indicates the presence of processes involving group identity and affection (Mason, 2018).

However, there are four findings that suggest that there are important limits to ideological polarization in the form of escalating divides between groups (as our Hypothesis 1 predicted). First, we can observe an increase in opposition to COVID measures on the left at the end of our study period. Second, right identifiers' opposition reaches a relatively stable level at the end of our study period. Third, the majority of respondents in each group believe that the current measures are appropriate. Fourth, all groups do only rarely respond that COVID measures are “not sufficient at all.”

The first and second of those findings lead to a convergence of positions toward COVID measures between all ideological groups at the end our observation period. This suggests that polarizing social influence on policy attitudes only persists if the political context stays stable. Dynamics can change greatly when the public attention to previously salient political topics fades (Baldassarri and Bearman, 2007) or when changes in the material realities that underly opinion polarization occur. In the final period, when COVID became politically manageable, vaccines were available and individuals had come to terms with the existence of the virus, the polarizing potential of COVID seems to slowly disappear.

Another account that is in line with all four findings is that ideologues of all camps orient themselves toward a global societal consensus when forming their political attitudes. As long as a significant share of individuals in the population holds centrist views, it is unlikely that even the more ideologically consistent groups radicalize in large parts. A related argument is that social influence networks are often not segregated to an extent that suffices to cause escalating polarization (Prior, 2013; Cardenal et al., 2019). These arguments can explain why the majority of each group believes that the current measures are appropriate throughout the pandemic. In addition, even the more pro-containment left-wing identifiers only rarely respond that COVID measures are “not sufficient *at all*.” This suggests that distancing from other opinions was not intense enough to lead left-wing identifiers to demand really extreme state restrictions. Orientation toward a global consensus might also explain left identifiers' slow trend toward more skepticism at the end of the study period, in which they seem to follow the center.

There are several limitations of this study. A first set of limitations concerns our use of left-right self-identification to distinguish ideological groups. These groups might not be fine-grained enough to capture the types of affinities and social influence processes that are necessary to lead to radicalization on the issue of COVID. It might be that the five broad ideological groups in this study mask extreme camps within the two poles. For example, it could be that the alternative, esoteric parts of the new left (adherents of new age spirituality or vaccine skeptics) (Frei and Nachtwey, 2022) and the alt-right parts of the right (who associate with the right-wing populist FPÖ, Mellacher, 2020) drift away from the center, while the rest of the left and right are rather moderate concerning COVID. Indeed, this could explain our finding of a limited escalation among right-wing identifiers: while FPÖ voters oppose COVID policies (Mellacher, 2020), the remaining right-wing identifiers might stay less opposed. This suggests that the radicalization potential is limited to only one subgroup in the right camp and does not spread to other subgroups. In contrast, the support for COVID policies on the left could be explained by negative influence (distancing from FPÖ supporters) and social influence among leftists. The probably most important limitation of our study is that the data come from an online access panel. Apart from the usual bias toward younger respondents, this might also bias our results if taking part in online surveys is associated with views on COVID. Our study shares this caveat with other studies on COVID related issues. Thus, there is a need for studies with common random samples in order to generalize our results to the wider population.

Overall, our study shows that ideological groups polarized in their opinion on the right policy reactions to the COVID pandemic. However, our results also show that polarization dynamics do not necessarily lead to escalating divergence of ideological groups. Rather, changing material conditions, the fading salience of political issues, and a consistently held centrist position by the majority put limits to the reinforcing polarizing processes of social influence.

## Data availability statement

Publicly available datasets were analyzed in this study. The datasets analyzed for this study can be found in the Austrian Social Science Data Archive, doi: https://doi.org/10.11587/28KQNS. More information about the data set used can be obtained at https://viecer.univie.ac.at/coronapanel/. Stata code will be made available on the author's Open Science Framework homepage upon publication (10.17605/OSF.IO/TB2WX).

## Author contributions

SD-S prepared the data, performed the statistical analysis, and wrote this draft of the manuscript.

## Funding

The position of SD-S was funded by the German Research Foundation as part of the project Opinion Polarization on Identity Politics and Denationalization Issues: A Longitudinal Comparative Perspective—Project Number: 440923825. We acknowledge support by the Open Access Publication Fund of the Freie Universität Berlin.

## Conflict of interest

The author declares that the research was conducted in the absence of any commercial or financial relationships that could be construed as a potential conflict of interest.

## Publisher's note

All claims expressed in this article are solely those of the authors and do not necessarily represent those of their affiliated organizations, or those of the publisher, the editors and the reviewers. Any product that may be evaluated in this article, or claim that may be made by its manufacturer, is not guaranteed or endorsed by the publisher.

## Footnotes

^1^We use the term ideological *group* polarization for two reasons. First, it stresses that the causes of polarization are not only related to identity as individuals perceive it, but also about the structure of the influence networks that individuals find themselves in, i.e., the ties in the social group (see next section). Second, it stresses that this paper is interested in broad group comparisons in contrast to fleshing out the attitudinal contents of certain ideologies.

^2^For detailed information, see https://viecer.univie.ac.at/coronapanel/austrian-corona-panel-data/method-report/.

^3^A more detailed, but still concise, history of COVID-related events in Austria can be found at https://viecer.univie.ac.at/en/projects-and-cooperations/austrian-corona-panel-project/corona-blog/corona-blog-beitraege/blog51/, and the following blog posts.

^4^ https://viecer.univie.ac.at/corona-blog/corona-blog-beitraege/blog112/
