# Supplementary material for: Ideological polarization during a pandemic: Tracking the alignment of attitudes toward COVID containment policies and left-right self-identification
Source: Front Sociol. 2022 Oct 28;7:958672. doi: 10.3389/fsoc.2022.958672 (PMC9650092; doi:10.3389/fsoc.2022.958672)
Supplement: Supplementary file 1 [file Data_Sheet_1.pdf]

Supplementary Material to ‘Ideological polarization during a pandemic: Tracking the alignment of attitudes towards COVID containment policies and left-right self-identification’

Supplementary Tables

Table S1: Descriptive sample statistics: Mean values of main variables in wave 1, wave 10, wave 15 and wave 20 (out of 24 waves). A refreshment sample in wave 10 leads to more cases in wave 10 than in the first wave. After wave 10, no new respondents enter our sample of analysis due to the operationalization of the left-right indicator. The same weights as those in the main analysis were applied.

	1	10	15	20
	mean	mean	mean	mean
Outcome variable				
Not sufficient at all	0.030	0.0026	0.034	0.042
Rather not sufficient	0.13	0.029	0.19	0.17
Appropriate	0.70	0.71	0.55	0.45
Rather too extreme	0.085	0.14	0.12	0.16
Too extreme	0.050	0.12	0.11	0.18
Left-right self-placement				
Left	0.14	0.15	0.14	0.14
Center left	0.20	0.20	0.20	0.20
Center	0.36	0.35	0.34	0.36
Center right	0.20	0.23	0.24	0.22
Right	0.085	0.075	0.073	0.076
Control variables				
Age	47.3	47.1	48.7	48.8
Ed. Primary ed. or less	0.19	0.20	0.19	0.19
Ed. Vocational training	0.47	0.45	0.46	0.47
Ed. Upper secondary ed.	0.17	0.18	0.18	0.17
Ed. University	0.17	0.18	0.18	0.17
Gender: Male	0.52	0.52	0.53	0.54
Gender: Female	0.48	0.48	0.47	0.46
Region: Vorarlberg	0.051	0.042	0.041	0.044
Region: Tirol	0.085	0.084	0.095	0.099
Region: Salzburg	0.054	0.060	0.051	0.050
Region: Steiermark	0.14	0.14	0.14	0.13
Region: Kaernten	0.069	0.066	0.059	0.064
Region: Oberoesterreich	0.17	0.16	0.17	0.18
Region: Niederoesterreich	0.19	0.20	0.20	0.19
Region: Wien	0.22	0.22	0.21	0.20
Region: Burgenland	0.029	0.034	0.037	0.035
Access balcony: no	0.44	0.48	0.47	0.48
Access balcony: yes	0.56	0.52	0.53	0.52
Access garden: no	0.44	0.42	0.43	0.45
Access garden: yes	0.56	0.58	0.57	0.55
Medical precondition: yes, one	0.16	0.15	0.15	0.15
Medical precondition: yes, several	0.067	0.072	0.082	0.077
Medical precondition: none	0.77	0.77	0.77	0.77
Observations	1152	1288	1059	1020

Table S2: Regression models used in main analyses and robustness analyses. Ordered logistic regression model coefficients in log odds scale. Ordered logit standard errors are clustered at the individual level. Table continues on the following pages.

	(1) Ordered logit, no controls (see Figure S1)		(2) Ordered logit with controls, not weighted (see Figure S2)		(3) Ordered logit with controls and weighted (main model, see Figure 2)		(4) Linear mixed model with random intercepts and random slopes (see Figure S3)	
Time variables								
Days after Feb. 25 (spline term 1)	0.00841***	(3.54)	0.0105***	(3.57)	0.00932**	(2.62)	0.00518***	(5.38)
Days (spline term 2)	-0.125***	(-4.50)	-0.145***	(-4.53)	-0.144***	(-3.47)	-0.0644***	(-6.38)
Days (spline term 3)	0.247***	(4.19)	0.285***	(4.22)	0.296***	(3.35)	0.127***	(6.08)
Days (spline term 4)	-0.161**	(-2.76)	-0.193**	(-3.00)	-0.232**	(-2.65)	-0.0910***	(-4.76)
Left-right self-placement, ref.: left								
Center left	0.0847	(0.41)	0.0156	(0.10)	0.00894	(0.04)	0.0604	(0.86)
Center	0.0439	(0.23)	0.0171	(0.11)	0.0738	(0.35)	0.132*	(2.04)
Center right	0.337	(1.47)	0.217	(1.22)	0.255	(1.06)	0.114	(1.62)
Right	-0.116	(-0.40)	-0.0509	(-0.20)	-0.0338	(-0.11)	0.0233	(0.26)
Interaction between time and left-right self-placement								
Center left X Days (spline term 1)	-0.00181	(-0.59)	-0.00164	(-0.67)	-0.00143	(-0.46)	-0.00160	(-1.92)
Center X Days (spline term 1)	0.00274	(0.94)	0.00135	(0.56)	0.00140	(0.46)	-0.000904	(-1.19)
Center right X Days (spline term 1)	0.000627	(0.19)	0.0000794	(0.03)	0.000646	(0.19)	-0.000379	(-0.46)
Right X Days (spline term 1)	0.00770	(1.78)	0.00686	(1.77)	0.00634	(1.46)	0.00260*	(2.50)
Center left X Days (spline term 2)	0.0150	(0.40)	0.0218	(0.80)	0.00760	(0.20)	0.0179*	(2.07)
Center X Days (spline term 2)	-0.0275	(-0.82)	-0.0169	(-0.65)	-0.0163	(-0.46)	0.00576	(0.74)
Center right X Days (spline term 2)	-0.0404	(-1.07)	-0.0196	(-0.68)	-0.0428	(-1.11)	-0.00224	(-0.27)
Right X Days (spline term 2)	-0.0407	(-0.92)	-0.0370	(-1.01)	-0.0253	(-0.55)	-0.0169	(-1.57)
Center left X Days (spline term 3)	-0.0209	(-0.26)	-0.0347	(-0.60)	-0.00289	(-0.03)	-0.0330	(-1.85)
Center X Days (spline term 3)	0.0740	(1.04)	0.0567	(1.03)	0.0542	(0.73)	-0.00441	(-0.27)
Center right X Days (spline term 3)	0.121	(1.50)	0.0690	(1.15)	0.127	(1.55)	0.0137	(0.79)
Right X Days (spline term 3)	0.0878	(0.93)	0.0877	(1.18)	0.0557	(0.57)	0.0369	(1.67)
Center left X Days (spline term 4)	-0.00571	(-0.07)	0.00601	(0.11)	-0.0271	(-0.32)	0.0197	(1.22)
Center X Days (spline term 4)	-0.107	(-1.53)	-0.0961	(-1.86)	-0.0965	(-1.34)	-0.0108	(-0.74)
Center right X Days (spline term 4)	-0.180*	(-2.27)	-0.115*	(-2.05)	-0.188*	(-2.37)	-0.0290	(-1.85)
Right X Days (spline term 4)	-0.100	(-1.10)	-0.111	(-1.64)	-0.0730	(-0.77)	-0.0379	(-1.90)

(continues)

Control variables:

Age (spline term 1)	0.0115	(1.74)	0.0218**	(2.75)	0.00609*	(2.43)
Age (spline term 2)	-0.0142	(-1.71)	-0.0286**	(-2.93)	-0.00406	(-1.30)
Education, ref.: primary ed. or less						
Vocational training	-0.104	(-0.54)	-0.200	(-0.85)	-0.0915	(-1.27)
Upper secondary ed.	0.252	(1.23)	0.358	(1.35)	0.127	(1.58)
University	0.243	(1.06)	0.107	(0.39)	0.0952	(1.10)
Vocational training X Days (spline term 1)	0.00156	(0.58)	0.00302	(0.92)	0.000878	(1.03)
Upper secondary ed. X Days (spline term 1)	-0.00477	(-1.61)	-0.00557	(-1.55)	-0.00196*	(-2.04)
University X Days (spline term 1)	-0.00479	(-1.50)	-0.00311	(-0.82)	-0.00188	(-1.84)
Vocational training X Days (spline term 2)	-0.0106	(-0.36)	-0.0173	(-0.46)	-0.00558	(-0.62)
Upper secondary ed. X Days (spline term 2)	0.0349	(1.06)	0.0635	(1.49)	0.0162	(1.61)
University X Days (spline term 2)	0.0536	(1.57)	0.0530	(1.20)	0.0187	(1.75)
Vocational training X Days (spline term 3)	0.0125	(0.20)	0.0179	(0.22)	0.00726	(0.39)
Upper secondary ed. X Days (spline term 3)	-0.0774	(-1.11)	-0.144	(-1.56)	-0.0345	(-1.65)
University X Days (spline term 3)	-0.122	(-1.71)	-0.131	(-1.38)	-0.0425	(-1.91)
Vocational training X Days (spline term 4)	0.0101	(0.17)	0.0266	(0.33)	0.00310	(0.18)
Upper secondary ed. X Days (spline term 4)	0.0865	(1.32)	0.161	(1.76)	0.0348	(1.82)
University X Days (spline term 4)	0.130*	(1.97)	0.157	(1.68)	0.0466*	(2.30)
Gender, ref.: male						
Female	-0.0693	(-0.88)	-0.149	(-1.61)	-0.0809**	(-2.87)
Region, ref. Vorarlberg						
Tirol	0.126	(0.54)	0.175	(0.65)	0.100	(1.20)
Salzburg	-0.305	(-1.27)	-0.414	(-1.75)	-0.0420	(-0.47)
Steiermark	-0.143	(-0.63)	-0.124	(-0.52)	0.0803	(1.03)
Kaernten	0.0383	(0.15)	0.00299	(0.01)	0.151	(1.71)
Oberoesterreich	0.0642	(0.29)	-0.0171	(-0.08)	0.128	(1.68)
Niederoesterreich	-0.253	(-1.14)	-0.288	(-1.27)	0.0144	(0.19)
Wien	-0.658**	(-3.03)	-0.879***	(-4.13)	-0.0786	(-1.02)
Burgenland	-0.640*	(-2.00)	-0.656*	(-2.37)	-0.115	(-1.08)
Access to balcony, ref.: no						
Yes	-0.138	(-1.59)	-0.114	(-1.09)	-0.0398	(-1.28)
Access to garden, ref.: no						
Yes	-0.182*	(-2.04)	-0.242*	(-2.25)	-0.0414	(-1.26)
Medical preconditions, ref. one						
Yes, several	-0.267	(-1.17)	-0.00213	(-0.01)	-0.134*	(-2.00)
None	0.128	(1.04)	0.0640	(0.48)	0.0336	(0.82)

(continues)

Model specific parameters

Mixed Model Constant							2.657***	(19.31)
Ordered logit cut-off 1	-3.425***	(-20.71)	-2.924***	(-7.77)	-3.381***	(-7.58)		
Ordered logit cut-off 2	-1.378***	(-9.11)	-1.088**	(-2.94)	-1.322**	(-2.99)		
Ordered logit cut-off 3	1.813***	(11.77)	1.782***	(4.86)	1.965***	(4.50)		
Ordered logit cut-off 4	2.750***	(16.71)	2.761***	(7.57)	2.928***	(6.78)		
Mixed Models, random terms:								
sd(Days Spline 1)							0.00237***	(-224.43)
sd(Days Spline 2)							1.11e-08	(.)
sd(Days Spline 3)							0.000000113	(-0.18)
sd(Days Spline 4)							0.00784***	(-90.08)
sd(Intercept)							0.526***	(-31.33)
sd(Residual)							0.531***	(-130.54)
bic	63663.7		65011.0		62803.0		51962.9	
N	27385		27362		27362		27362	
Standard errors	clustered		clustered		clustered			

t statistics in parentheses
* $p < 0.05$, ** $p < 0.01$, *** $p < 0.001$

Supplementary Figures

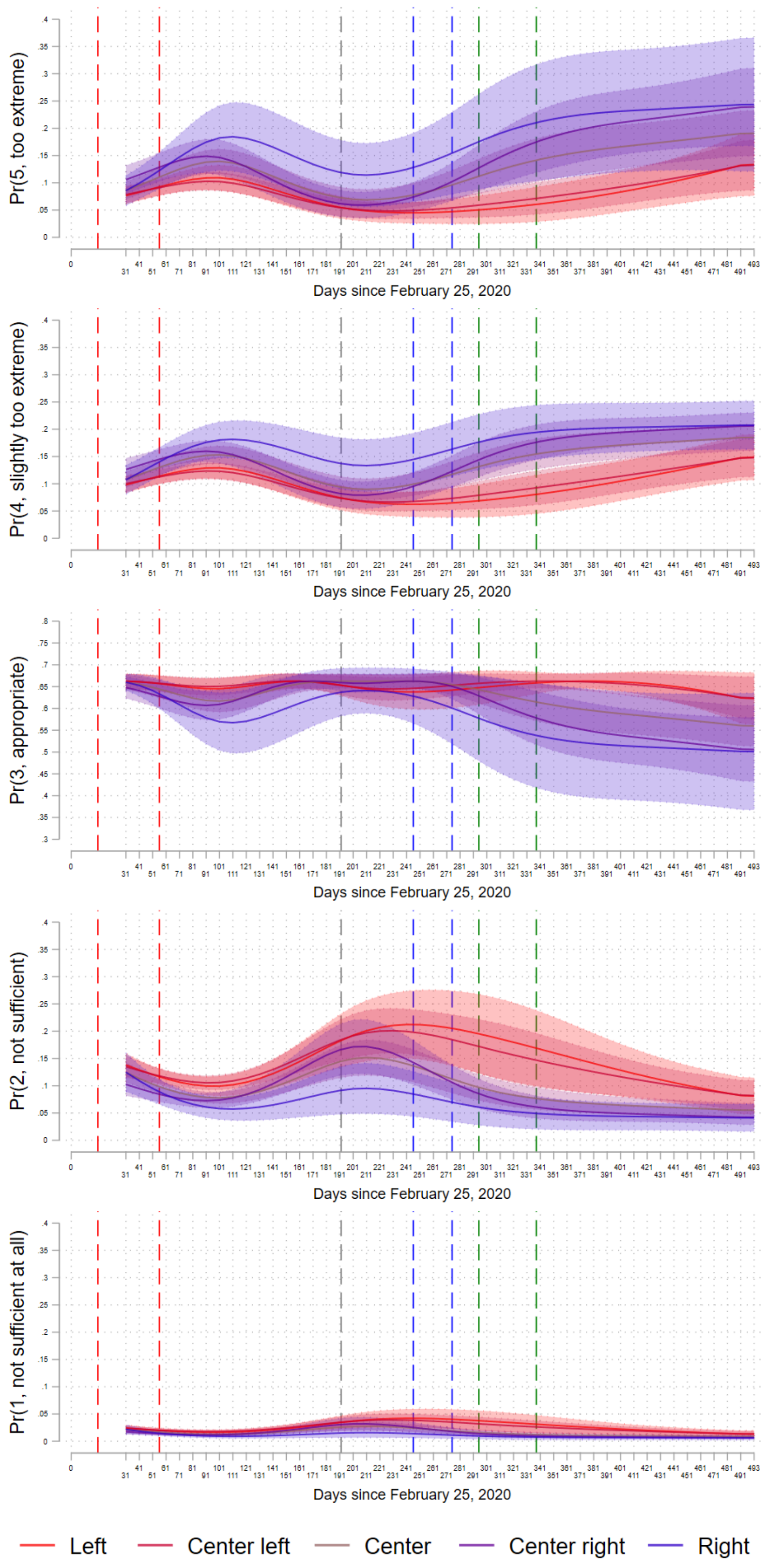

Figure S1: Results from a model similar to the main model, but without control variables. See Table A2, model 1.

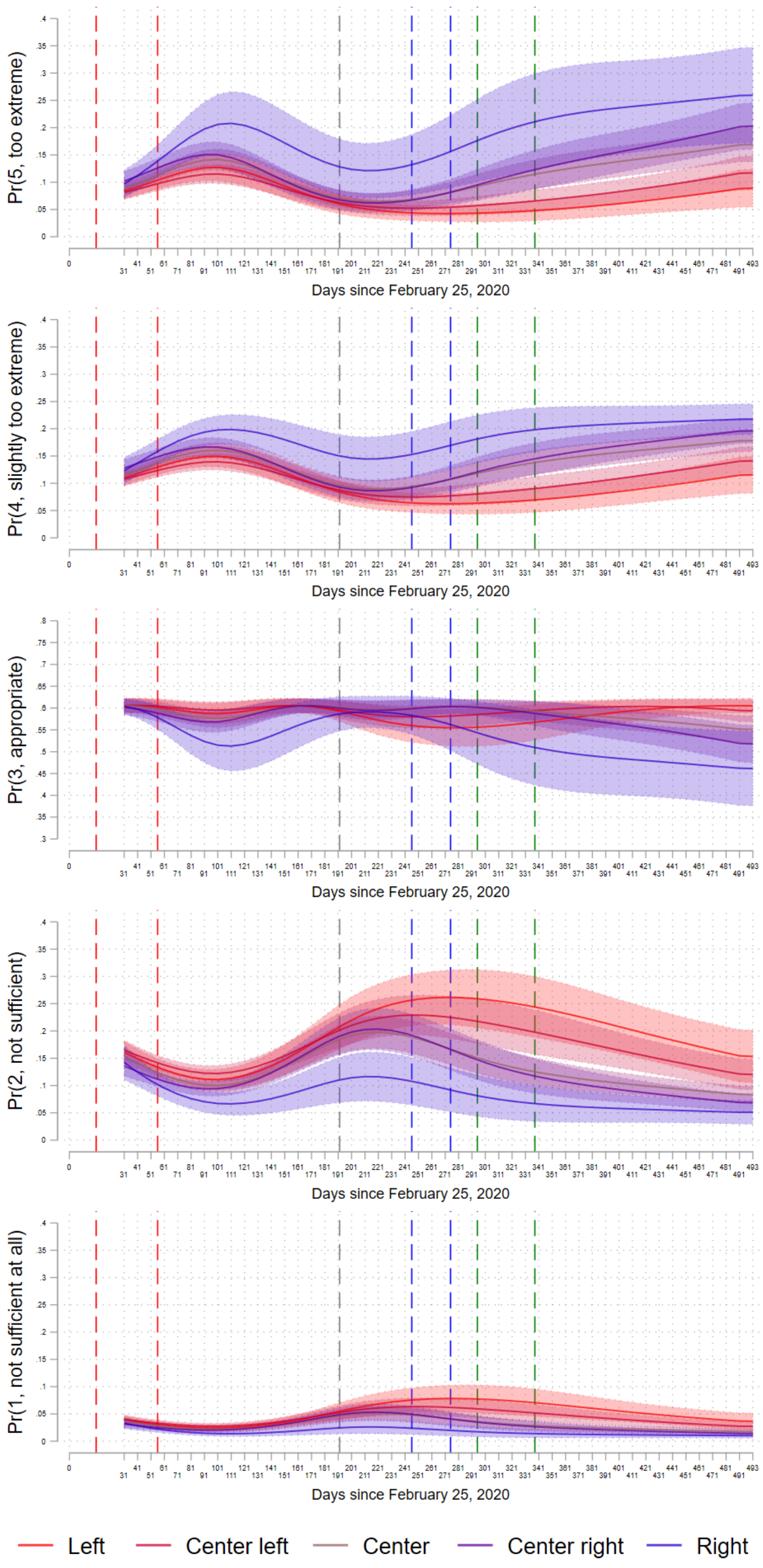

Figure S2: Results from a model similar to the main model with control variables, but without inverse-probability of censoring weights and without demographic and political weights. See Table A2, model 2.

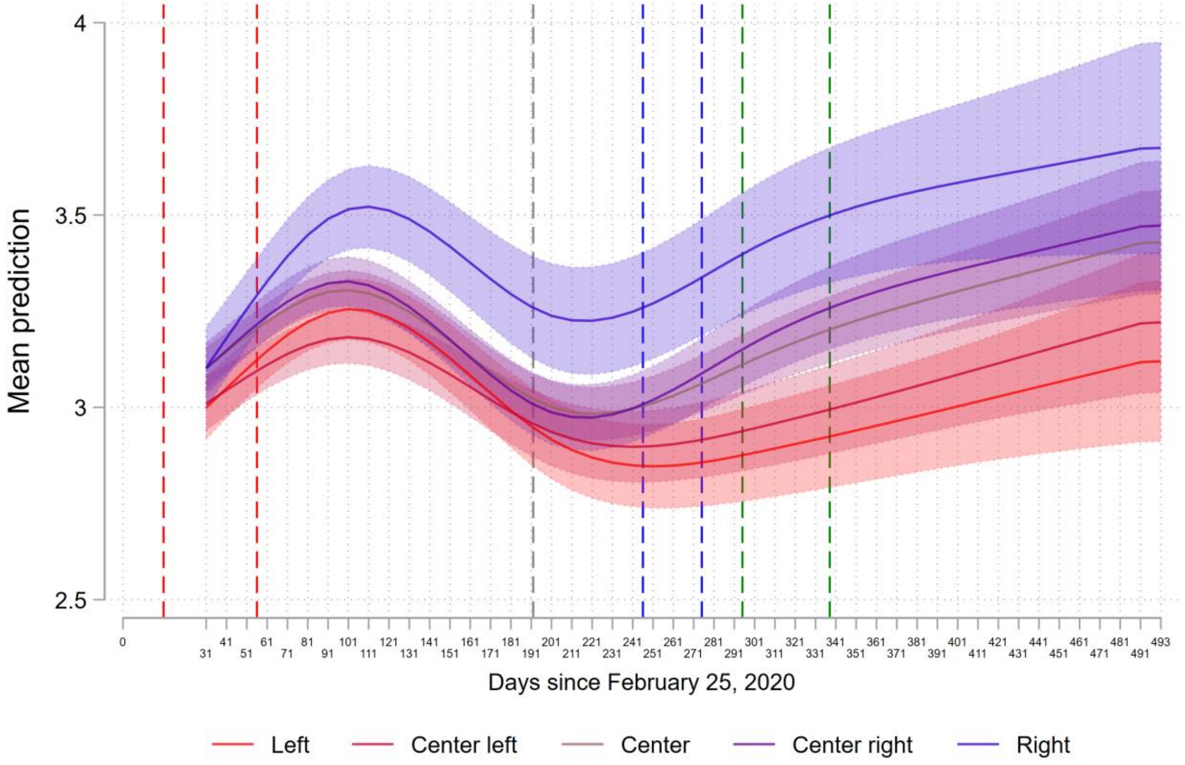

Figure S3: Another way to model time trends via Mixed Models (also known as Growth Curve Models): linear hierarchical model with random intercepts and random slopes for each spline term of time. See Table A2, model 4.

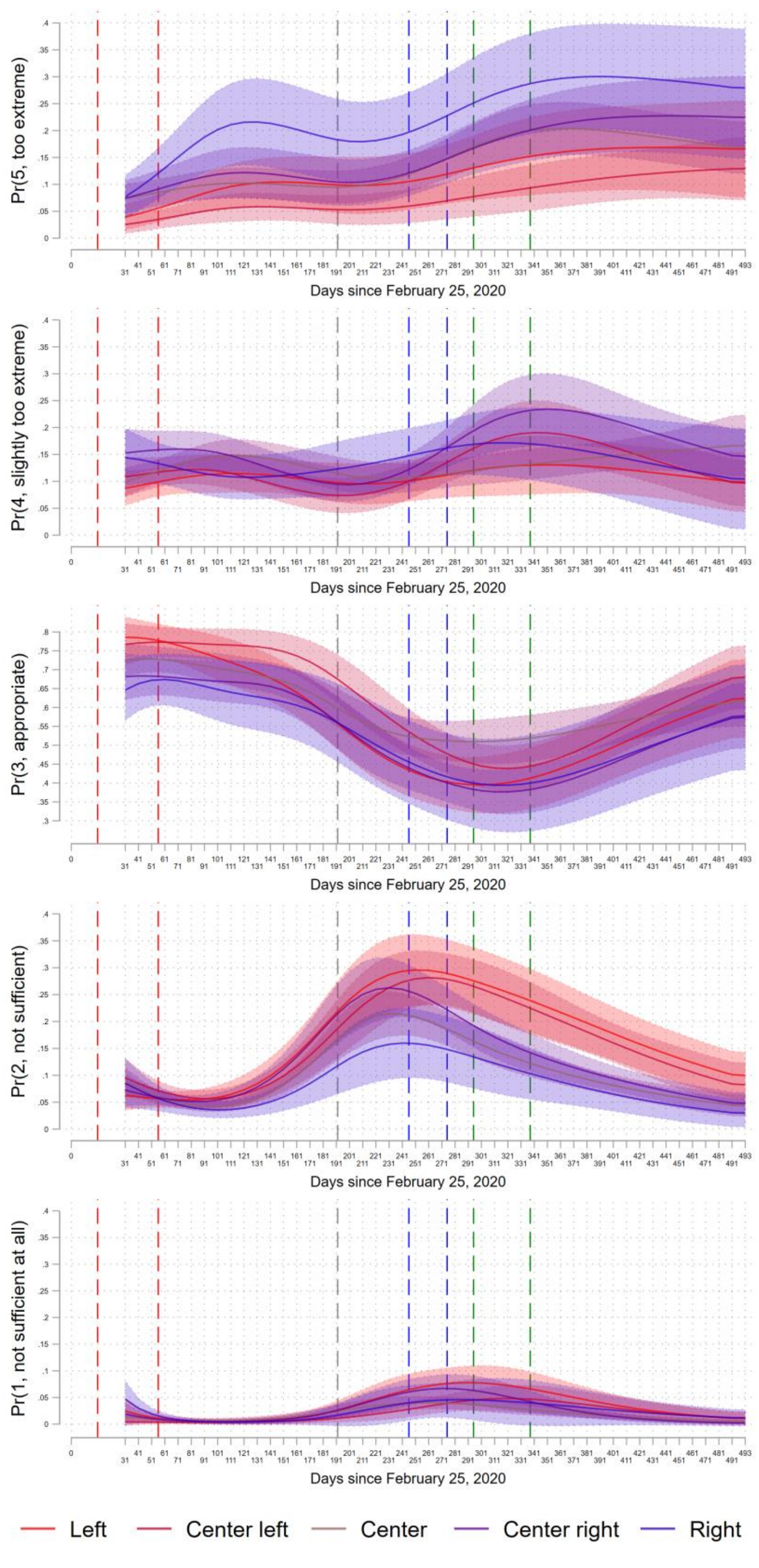

Figure S4: Results from multinomial logistic regression with control variables and weighted. Regression table is not shown because of its length, but is available upon request.
